# Anguillid eels as a surrogate species for conservation of freshwater biodiversity in Japan

**DOI:** 10.1038/s41598-020-65883-4

**Published:** 2020-05-29

**Authors:** Hikaru Itakura, Ryoshiro Wakiya, Matthew Gollock, Kenzo Kaifu

**Affiliations:** 1 Chesapeake Biological Laboratory, University of Maryland Center for Environmental Science, 146 Williams St., Solomons, MD 20688 USA; 2 Graduate School of Science, Kobe University, 1-1 Rokkoudaichou, Nadaku, Kobe, Hyogo 657-8501 Japan; 3 Research and Development Initiative, Chuo University, 1-13-27 Kasuga, Bunkyo-ku, Tokyo 112-8551 Japan; 4 Atmosphere and Ocean Research Institute, the University of Tokyo, 5-1-5 Kashiwanoha, Kashiwa, Chiba 277-8564 Japan; 5 The Zoological Society of London, Regent’s Park, London, NW1 4RY UK; 6 Faculty of Law, Chuo University, 724-1 Higashinakano, Hachioji-shi, Tokyo 192-0393 Japan

**Keywords:** Biodiversity, Conservation biology, Freshwater ecology

## Abstract

To monitor and manage biodiversity, surrogate species (i.e., indicator, umbrella and flagship species) have been proposed where conservation resources are focused on a limited number of focal organisms. Using data obtained from 78 sites across six rivers in the mainland Japan and the Amami-Oshima Island, we demonstrate that two anguillids – the Japanese eel (*Anguilla japonica*) and the giant mottled eel (*A. marmorata*) – can act as surrogate species for conservation of freshwater biodiversity. Anguillid eels were the widest topographically-distributed species ranging from near the mouth to the upper reaches of rivers. Moreover, stable isotopic analyses indicated that eels are likely one of the highest-order predators in freshwater ecosystems. A significant positive relationship was found between the density of eels and the number of other diadromous species collected. However, the optimal models revealed that both the density of eels and the number of other diadromous species were significantly negatively correlated with distance from the river mouth and cumulative height of trans-river structures from the river mouth to each site. This suggests the positive relationship between eel density and number of other diadromous species was indirect and related to river-ocean connectivity. Given their catadromous life-cycle, and global commercial and cultural importance, as a taxa, anguillid eels can act as indicator, umbrella and flagship species, and a comprehensive surrogate for conservation of freshwater biodiversity.

## Introduction

Although fresh waters cover only 2.3% of the Earth’s surface^1^, the number of described species per area is much higher than that of terrestrial and marine ecosystems^2^. Further, they support approximately 10% of all known species, which includes 40% of global fish species and ~33% of global vertebrate species^3,4^. However, declines in biodiversity are far greater in fresh waters than the most affected terrestrial ecosystems^5^. Freshwater ecosystems are the most globally threatened; they also concentrate human populations that have led to widespread habitat degradation, pollution, flow regulation, water extraction, unsustainable fisheries, alien species introductions, changing climates, infectious diseases, harmful algal blooms and expanding hydropower^6–8^. As a result, nearly one-third of species in fresh waters have been classified as ‘Endangered’ in the International Union for Conservation of Nature (IUCN) Red List of Threatened Species^9^.

Monitoring and managing all aspects of biodiversity is challenging, and ‘shortcuts’, such as a surrogate species (i.e., indicator, umbrella and flagship species), whereby resources are focused on a limited number of focal species for broader benefit^10^. Depending on the conservation goals, several concepts of surrogate species have been distinguished^11^. ‘Indicator species’ have been used to assess the magnitude of anthropogenic disturbance and changes in habitat (health indicators), to locate areas of high regional biodiversity (biodiversity indicators), and to monitor population trends in other species (population indicators)^12^; ‘umbrella species’ have been proposed as a way to manage entire communities by focusing on the requirements of the most widespread species^13^; ‘flagship species’ have been employed to attract public attention and support for nature at a global or national levels, and potentially attract funding for larger environmental issues^14^. Regardless of their underlying assumptions, the expectation is that the presence and/or abundance of a surrogate species is a means to understanding the composition, state and function of a more complex community, and the use of them has been thought to have merit for conservation and management of natural environments^10,15,16^. Many species have been proposed as surrogates in terrestrial (large mammals or birds)^17,18^ and aquatic ecosystems (mammals or fish)^19–21^, but the assumed functional or distributional relationships between these and other taxa are rarely tested^22^. Although freshwater surrogate species, including migratory species, have been considered conceptually, they are rarely used in aquatic ecosystems^16,23^.

Recently, it was proposed that catadromous eels (genus *Anguilla*), including 16 species that spawn in the open ocean and grow in continental waters, are promoted as a flagship species for aquatic conservation^24^. This was proposed on the grounds that (1) stocks of some anguillid eels (hereafter referred to as eels) have experienced remarkable declines in recent decades, which has led to 10 of 16 species assessed now being listed as ‘Threatened’ or ‘Nearly Threatened’ in the IUCN Red List of Threatened Species^25^; (2) they have catadromous life-cycles involving extended migrations through both marine and freshwater environments in more than 150 countries^26^; (3) threats such as climate change, barriers to migration, pollution, habitat loss, and unsustainable exploitation and trade threaten eels globally^27^, all of which will have significant impacts on thousands of other aquatic species that are resident in both marine and freshwater ecosystems^24^. Given the catadromous life-cycles of eels that have long fascinated researchers and their global commercial and cultural importance, they have the potential to stimulate public interest and support for conservation. Moreover, considering their ecological characteristics, broad habitat use extending from saline bays to upland headwaters^28^ as well as polytrophic feeding habits^29–33^, they have potential to be not only a flagship species, but also indicator and umbrella species for freshwater biodiversity. This could have huge benefits for other aquatic flora and fauna, many of which are even more poorly understood than eels^24^.

Here, we demonstrate that eels can be a surrogate species for conservation of freshwater biodiversity using data relating to aquatic species including both the Japanese eel (*A. japonica*) and the giant mottled eel (*A. marmorata*). The research was conducted in rivers in Japan, a part of islands formed by the accretionary prism^34,35^. It has been known that migratory diadromous species are generally predominant in rivers of the regions formed by the accretionary prism^36^, because many small rivers are formed in these regions as a result of having many mountainous areas compared to the eroded regions and the craton^34^. Since diadromous species recruit between marine and freshwater environments, trans-river structures such as dams and weirs have a critical impact on declines of population for diadromous species including eels^37^. Thus, such islands offer suitable sites to investigate possible effects of river-ocean connectivity on freshwater biodiversity.

The potential of using eels as surrogate species for conservation of freshwater faunal diversity was evaluated by testing (1) whether eels were the widest topographically-distributed species in freshwater ecosystems, (2) whether eels were appropriate indicator of river-ocean connectivity and (3) whether eels were a high-order consumer in freshwater ecosystems. Finally, we discussed the potential of eels to act as a comprehensive symbol for freshwater conservation by synthesizing the results of the present study and previous reports showing the commercial and cultural importance of eels.

## Methods

### Study species

*A. japonica* spawn in the waters west of the Mariana Islands located in the western North Pacific Ocean^38^, and they grow in a wide range of habitats within a river, from brackish estuaries to upland headwaters, lakes, and in saline bays^39,40^ in East Asia including Taiwan, eastern China, Korea, and Japan. *A. japonica* is a commercially important species in East Asia, and was classified as Endangered on the IUCN Red List of Threatened Species due to a notable decline in abundance^41^.

*A. marmorata* is the most widespread anguillid species in the world, from the western Indian Ocean, across the Indo-Pacific, to French Polynesia in the South Pacific Ocean^42,43^. They have four genetically different populations^44^, one of which spawns in same region with *A. japonica*^38^. Although *A. marmorata* tends to reside in freshwater areas rather than brackish or seawaters^45^, they grow in a wide range of freshwater habitats^46,47^.

### Study area

**Figure 1:**
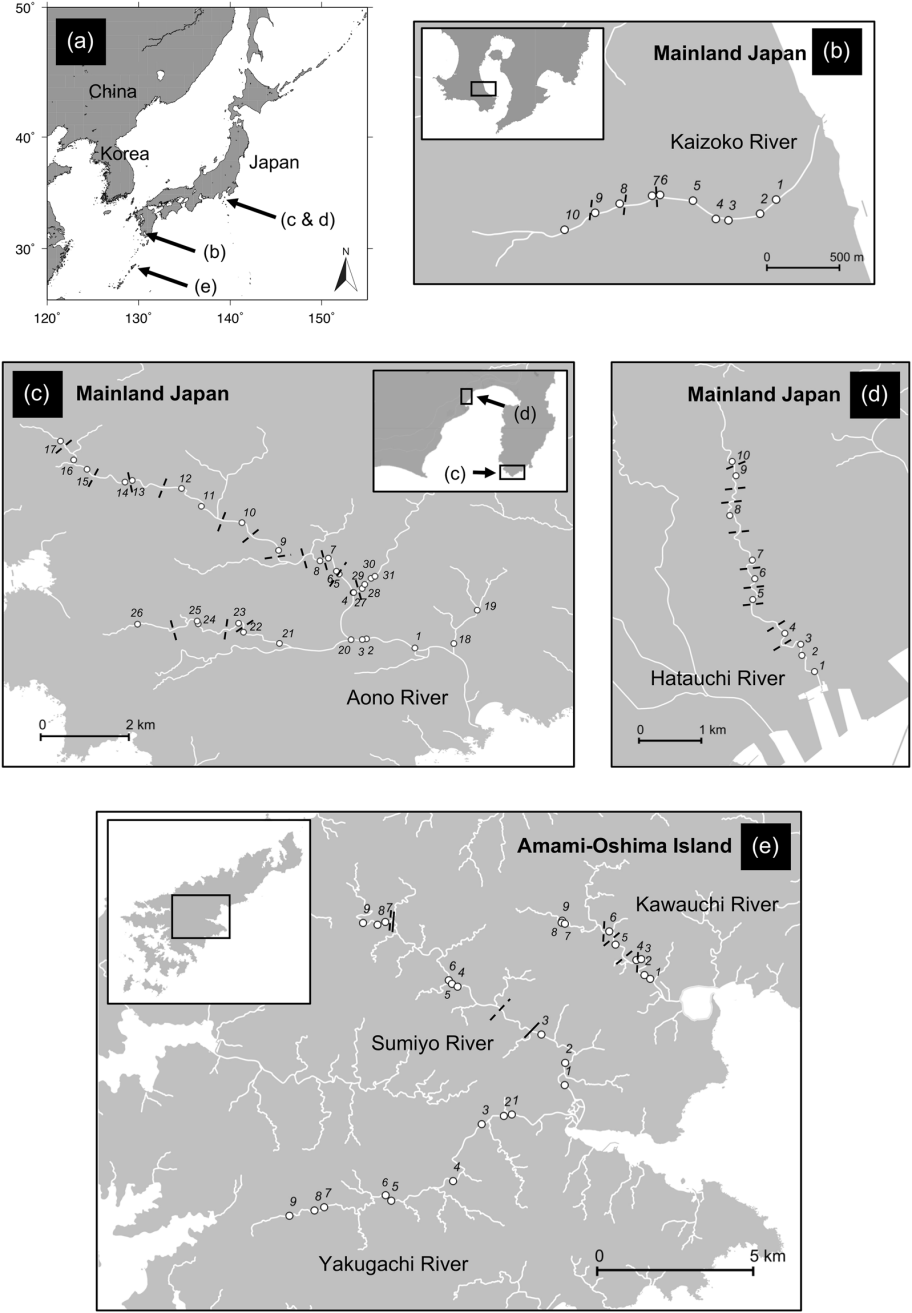
Maps of the study rivers and each study site in Japan where sampling for aquatic animals were conducted. Locations of the study rivers in Japan (**a**), the Kaizoko River (**b**), the Aono River (**c**), the Hatauchi River (**d**), and rivers in the Amami-Oshima Island (**e**). The white circles indicate locations of each study site, the numbers around which indicate the study site within each river. The thin and bold dashed lines indicate the presence of one or more weirs or dams, respectively, while the solid lines indicate waterfalls.

Because *A. japonica* and *A. marmorata* primarily inhabit rivers in the mainland Japan and subtropical islands (Nansei Islands) in Japan, respectively^39,47^, this study was conducted in six small rivers in the Kagoshima and Shizuoka Prefectures, located in these two different regions (Fig. 1a): mainland Japan, which includes the central main island of Japan (the Aono and Hatauchi rivers; Fig. 1c,d) and the southern main island of Japan (Kaizoko River; Fig. 1b); and Amami-Oshima Island, which is a subtropical island (the Kawauchi, Sumiyo and Yakugachi rivers; Fig. 1e). Amami-Oshima is believed to be near the northern limit of the distribution range of *A. marmorata*^48^. Each of these rivers has a length of <20 km and a basin area of <50 km^2^ (Table 1). We chose these because (1) such rivers allowed us to conduct quantitative sampling throughout freshwater areas using electrofishing, and (2) there were no commercial fishery or eel stocking activities in them, providing good model systems to test our hypotheses. All rivers studied flow through agricultural and forest lands. A total of 78 study sites were distributed throughout the full length of each river (9–31 sites per river; Fig. 1b–e, Table 1). The study sites were split into three equal parts in the mainstream of Aono River (lower, middle and upper reaches: site number 1–6, 7–12 and 13–17, respectively; Fig. 1c) and rivers in the Amami-Oshima Island (lower, middle and upper reaches for each river: site number 1–3, 4–6 and 7–9, respectively; Fig. 1e), to estimate the trophic level of animals captured in each reach.

**Table 1:** Characteristics of the study rivers and sampling sites of freshwater species in Japan.

River	Region	Prefecture	Length (km)	Basin area (km^2^)	Study period	Study sites		Trans-river structure	
						No.	Length (m)	No.	Height (m)
Hatauchi	Mainland Japan	Shizuoka	4.3	8	Sep. 2016	10	40	40 (0)	0.9 ± 0.7 (0.1–3.4)
Aono	Mainland Japan	Shizuoka	17.2	72	Sep.–Oct. 2015	31	18.5 ± 2.4 (12–20)	27 (0)	1.2 ± 0.7 (0.2–2.6)
Kaizoko	Mainland Japan	Kagoshima	3	—	Aug. 2016	10	40	7 (0)	1.0 ± 0.4 (0.2–1.4)
Yakugachi	Amami-Oshima Island	Kagoshima	15.1	45.1	Aug.–Sep. 2015	9	19.6 ± 1.3 (16–20)	0 (0)	0
Sumiyo	Amami-Oshima Island	Kagoshima	16.8	48.5	Aug.–Sep. 2015	9	20	4 (2)	18 ± 11.5 (5.0–30.0)
Kawauchi	Amami-Oshima Island	Kagoshima	11.6	41.7	Aug.–Sep. 2015	9	20	4 (0)	2.4 ± 0.4 (2.0–2.8)

Length of study sites and height of trans-river structure are shown by mean ± SD and range (in parentheses). Values in parentheses of no.of trans-river structure indicate no. of waterfall.

### Field sampling

We carried out field sampling from August 2015 to September 2016 (Table 1). Freshwater species were collected from each study site using a battery-powered backpack electrofishing unit operating at 200-V DC (LR-20B; Smith-Root, Inc., Vancouver, WA, USA). At each study site, all captured animals were identified to species level. We only used fish and crustacean species, because other animals such as amphibians and aquatic insects were rarely collected in this study (see Results). They were further classified into diadromous and non-diadromous species. In the Kaizoko and Hatauchi rivers, eels were anaesthetised with 10% eugenol solution (FA100; DS Pharma Animal Health Co., Ltd. Japan) in the field before the measurements, and then they were returned to capture sites for another study. In contrast, collected eels in other rivers were euthanised with >10% eugenol solution before being stored at −20 °C, and then dissected for stable isotope analysis. A topographical distribution of each species within each river was calculated by dividing the distance between the highest and lowest study sites of each river where each animal was collected, by the distance between the highest and lowest study sites of each river. The coverage was averaged by each region (the mainland Japan and Amami-Oshima Island).

Environmental conditions at each study site were taken following the sampling (Table 1; Supplementary Table S1). The depth and velocity were measured at the center of the downstream, middle and upstream points at each study site and the mean of the three measurement points were used for analysis. The sediment was categorised into three types: (a) mud, sand or gravel; (b) concrete or bedrock; and (c) whether boulders that can provide refuges for eels were present. The presence of riparian vegetation was also noted. The height of all trans-river structures such as weirs, dams and waterfalls that were found from the river mouth to the most upstream site of each river was measured and the cumulative height (hereafter referred to as ΣhTRS) was calculated. We found a total of 82 trans-river structures in the study rivers, and their height ranged from 0.1 m to 30.0 m (mean ± SD = 1.9 ± 4.3 m, median = 1.0 m), while no trans-river structure was found in the Yakugachi River (Fig. 1e). Large dams (>15 m in height) and waterfalls were only found in the Sumiyo River (Fig. 1e).

### Stable isotope analysis

All animals captured in the mainstream of Aono River and rivers in the Amami-Oshima Island were used for stable isotope analysis in order to estimate their trophic levels. Muscle tissues of these animals were used as it has slow turnover rate, resulting in a history of food assimilation over a period of months, thereby excluding short-term variability^49^. All samples were dried in an oven at 60 °C for 24–48 h, and ground to a fine powder using a mortar. Then, 0.5 to 1.0 mg of each ground sample was sealed into a tin capsule. Nitrogen stable isotope ratio was analyzed using an elemental analyzer (FLASH 2000, Thermo Electron, Italy) interfaced with a mass spectrometer (Delta V advantage, Thermo Finnigan, Germany) via a ConfloIV open split interface (Thermo Finnigan, Germany). The isotope ratios were expressed as per thousand (‰) deviation according to the international standard of atmospheric N_2_ in which δ^15^N = (^15^N/^14^N_sample_/^15^N/^14^N_standard_ − 1) × 1.000. The error of measurement was within ± 0.15‰.

The trophic level (TL) of all captured animals was calculated using the following equation: TL = [(δ^15^N_consumer_ − δ^15^N_base_)/Δ δ^15^N] + 2, where δ^15^N_consumer_ is the δ^15^N of the consumer, δ^15^N_base_ is the baseline δ^15^N value of the food web, ∆ δ^15^N is the trophic enrichment factor (TEF), and the value 2 indicates the TL of the organism used to establish the δ^15^N_base_. When TLs of eels were calculated, the TEF was set at 2.1‰ per TL following Kaifu *et al*.^30^ that estimated unique TEF values of reared yellow-phase Japanese eels. By contrast, when TLs of other animals were calculated, it was set at 3.4‰^50^. Japanese eels inhabiting rivers primarily belong to the littoral food web^30^. Thus, for δ^15^N_base_, we used mean δ^15^N of the rock climbing goby *Sicyopterus japonicus* collected in each reach of each river as the end-member of the littoral food web, because this species is a primary consumer (TL = 2) feeding mainly on algae^51^, which has protracted isotopic turnover rates integrating spatial-temporal variability. Indeed, δ^15^N of this species was lower than that in other species in this study.

### Statistical analysis

All statistical analyses were performed with R 3.6.0. To evaluate the possibility of eels as an indicator of river-ocean connectivity, the relationship between density of eels and number of other diadromous species was tested. The other diadromous species did not include species that lives entirely in fresh waters. We used a generalized linear mixed model (GLMM; *glmer.nb* in the package *lme4*)^52^, which included the number of eels as a response variable, number of other diadromous species and eel species (i.e., *A. japonica* or *A. marmorata*) as the explanatory variables, and area (m^2^) at each study site as an offset term. To assess the relationship between density of eels and environmental factors, we also used the GLMM, which included number of eels as a response variable, environmental factors and eel species as the explanatory variables, and area (m^2^) at each study site as an offset term. We also assessed the relationship between number of other diadromous species and environmental factors using the GLMM, which included number of other diadromous species as the response variable and environmental factors as the explanatory variable. The environmental factors included sediment (quantitative variable, i.e., a, b or c), depth, distance from the river mouth, ΣhTRS from the river mouth, vegetation (quantitative variable, i.e., 1 or 0), and water velocity. To avoid multicollinearity of environmental variables, we first checked the correlations between each pair of environmental variables using Pearson’s correlation test and confirmed that all pairs were not highly correlated variables (*r* < 0.6). A negative binomial distribution was used for the response variables of all models with a log-link function. All models included the river as a random effect. In the models that included environmental factors, we constructed the models including all explanatory variables and the models that yielded the lowest Akaike’s information criterion (AIC); those with ∆AIC < 2 were selected for descriptive purposes^53^. Model selections were performed using *dredge* in the package *MuMIn*^54^. After selecting the lowest AIC model (i.e., best model), whether zero included in 95% confidence interval of the coefficients (Wald statistics) of explanatory variables that were selected by the best model, i.e., the statistical significance of selected explanatory variables was evaluated using Wald tests.

It was expected that effects of trans-river structure on movement of aquatic animals differed depending on its height; for example, the effects of a structure 1 m in height and 10 successive structures of 10 cm in height on movement of animals should be different, but both of them were calculated to be 1 m of ΣhTRS, which have an identical effect on aquatic animals in the model used in this study. Prior to assessing the relationship between density of eels and environmental factors, therefore, we tested the lower limit of the height of trans-river structure that should be included in the calculation of ΣhTRS. In this analysis, we used the GLMM, which included number of eels as a response variable, ΣhTRS as an explanatory variable, area (m^2^) at each study site as an offset term, and the river as a random effect. We assessed 31 candidate models having different ΣhTRS that were calculated by varying the lower limit value of the height of the structure from 0 to 300 cm by 10 cm, using AIC.

TLs of eels and other species in each reach of each river were compared using Wilcoxon rank sum test (*wilcox.exact* in the package *exactRankTests*)^55^. Additionally, the magnitude of differences of TLs between eels and other species was quantified using Cliff’s delta effect size statistics (|*d* | ; *cliff.delta* in the package *effsize*)^56^. In this study, we considered |*d* | >0.33 as a threshold for significance following Romano *et al*.^57^.

### Ethical statement

All experiments including the sampling were conducted under the relevant guidance and regulations with the permission of the Fisheries Adjustment Rules of Kagoshima and Shizuoka Prefectures, and our protocols were approved by Institutional guidelines for animal experiments of Chuo University. The sampling efforts have been made to minimize the number of animals killed for this study.

## Results

### Number of collected eels and freshwater species

A total of 216 individuals of the two eel species were collected in this study; 129 *A. japonica* were collected in rivers of the mainland Japan and 87 *A. marmorata* were collected in rivers of the Amami-Oshima Island (Table 2). The total length of *A. japonica* ranged from 91 to 609 mm with a mean ± SD of 327 ± 129 mm, and that of *A. marmorata* ranged from 119 to 1320 mm with a mean ± SD of 363 ± 171 mm (Table 2). In addition, we collected a total of 48 fish and crustacean species, which included 36 species in the mainland Japan, 21 species in the Amami-Oshima Island and 9 species in both regions (Supplementary Table S2). Of the collected species, 80% (78.4% at mainland Japan and 90.9% at Amami-Oshima Island) were classed as a diadromous species (Supplementary Table S2). As a total of four amphibian and aquatic insect species were collected only in the Aono River of the mainland Japan, we excluded these species from the analyses.

**Table 2:** Summary of the collected specimens in Mainland Japan and Amami-Oshima Island, Japan.

River	Eel species	Anguillid eels			No. of other diadromous species
		No. of eels	Total length (mm)	Body weight (g)	
Hatauchi	*A. japonica*	11	361 ± 108 (201–544)	72 ± 66 (8–215)	9 ± 3 (4–14)
Aono	*A. japonica*	70	339 ± 140 (91–599)	82 ± 93 (0.83–339)	6 ± 2 (2–11)
Kaizoko	*A. japonica*	48	301 ± 119 (110–609)	50 ± 62 (2–304)	7 ± 3 (4–12)
Yakugachi	*A. marmorata*	35	329 ± 120 (148–615)	107 ± 143 (4–619)	6 ± 2 (4–9)
Sumiyo	*A. marmorata*	14	453 ± 271 (200–1320)	703 ± 1988 (16–7580)	3 ± 2 (1–7)
Kawauchi	*A. marmorata*	38	361 ± 153 (119–766)	186 ± 258 (2–1310)	6 ± 3 (3–13)

Total length, body weight, and no. of other diadromous species are shown by mean ± SD and range (in parentheses).

### Distributional range

*A. japonica* had the widest distributional coverage of all captured animals, which covered 86.5% of the range of the study rivers in mainland Japan (Table 3). *A. marmorata* had the widest distribution in the study rivers of the Amami-Oshima Island at 93.7% of the range (Table 3).

**Table 3:** A topographical distribution coverage (%) of each animal at Mainland Japan and Amami-Oshima Island, Japan.

Region	Species	Common name	Migratory type	Coverage (%)
Mainland Japan	*Anguilla japonica*	Japanese eel	Diadromous	86.5 (59.6–100.0)
	*Eriocheir japonica*	Japanese mitten crab	Diadromous	84.2 (61.1–100.0)
	*Macrobrachium japonicum*	Freshwater prawn	Diadromous	69.2 (66.7–71.9)
	*Gymnogobius petschiliensis*	Floating goby	Diadromous	62.3 (52.6–77.8)
	*Rhinogobius nagoyae*	Freshwater goby	Diadromous	57.6 (38.9–89.9)
	*Macrobracbium formosense*	Freshwater prawn	Diadromous	55.6 (13.5–88.9)
	*Plecoglossus altivelis altivelis*	Ayu	Diadromous	52.8 (27.9–77.8)
	*Tridentiger brevispinis*	Dusky tripletooth goby	Diadromous	35.4 (23.3–58.3)
Amami-Oshima Island	*Anguilla marmorata*	Giant mottled eel	Diadromous	93.7 (93.8–95.7)
	*Macrobrachium japonicum*	Freshwater prawn	Diadromous	80.3 (66.3–100.0)
	*Sicyopterus japonicus*	Rock climbing goby	Diadromous	72.5 (51.1–100.0)
	*Macrobracbium formosense*	Freshwater prawn	Diadromous	60.1 (25.5–100.0)
	*Tridentiger kuroiwae*	Dusky tripletooth goby	Diadromous	53.1 (17.0–87.4)
	*Plecoglossus altivelis ryukyuensis*	Ayu	Diadromous	43.6 (17.0–81.8)
	*Caridina multidentata*	Japanese marsh shrimp	Diadromous	19.5 (6.2–39.6)
	*Eleotris fusca*	Sleeper gobies	Diadromous	6.5 (0.0–19.6)

The coverage was shown by mean and range (in parentheses) values of each three rivers in each region. Note that only top eight species of each region were shown.

### Relationships between eels, other diadromous species and environmental conditions

In the 31 candidate models showing relationship between the density of eels and ΣhTRS, the AIC value of the model having ΣhTRS that was calculated by considering all heights of the structure (i.e., the lower limit of height was 0 cm) was the lowest (Supplementary Fig. S1). Thus, we employed all heights of trans-river structure to calculate the ΣhTRS.

**Figure 2:**
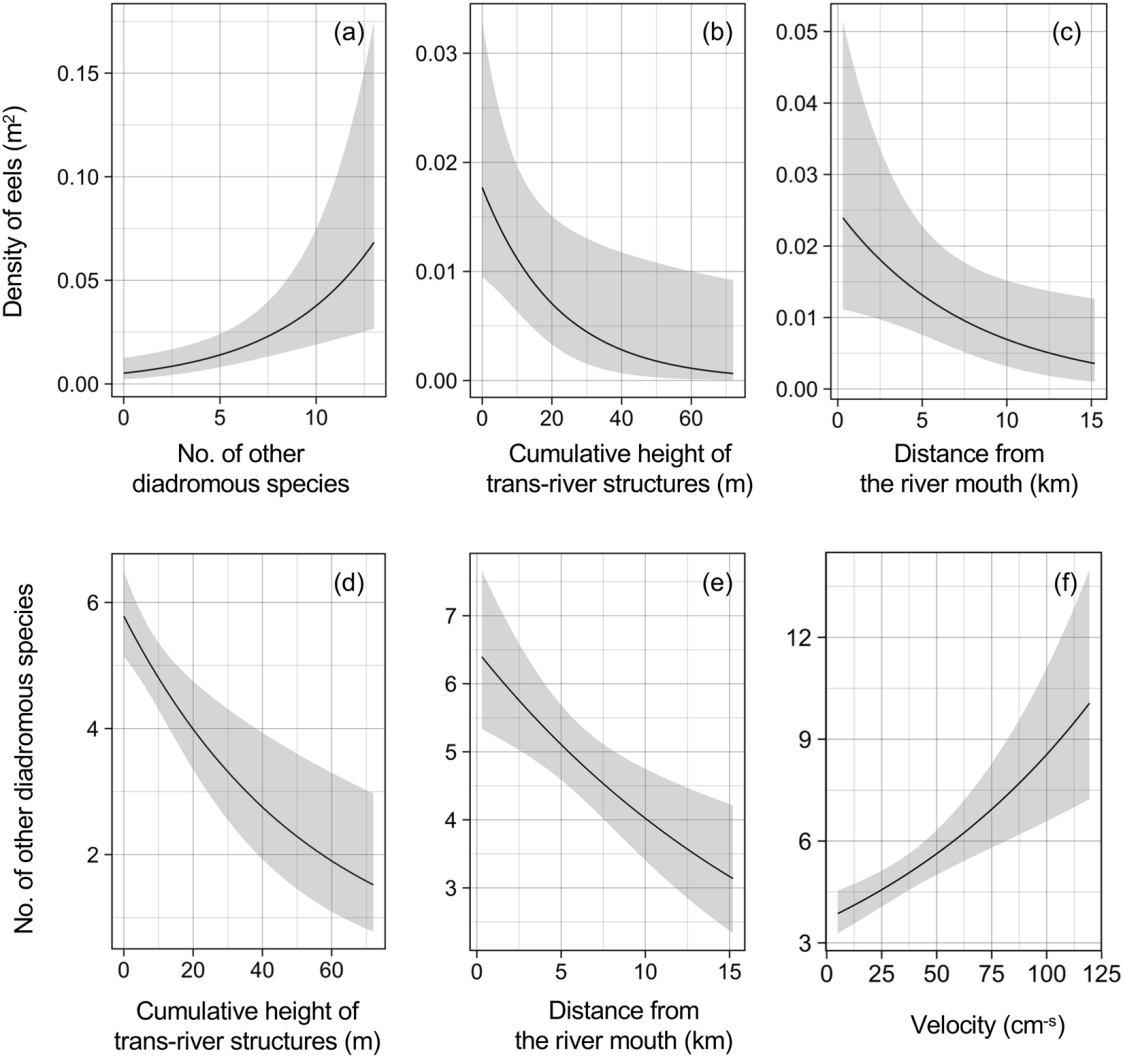
Graphic summaries of generalized linear mixed models assessed relationships between density of anguillid eels and number of other diadromous species (**a**); between density of anguillid eels and environmental factors (**b** and **c**); and between number of other diadromous species and environmental factors (**d**–**f**). The lines and shaded areas indicate the predictive value and 95% intervals of the models, respectively.

The estimated coefficient of GLMM showed that the density of eels was significantly positively correlated to the number of other diadromous species (coefficient ± SE = 0.198 ± 0.058, *z* = 3.408, *P* = 0.0007; Fig. 2a). By contrast, the density of eels did not vary between the two species (coefficient ± SE = 0.559 ± 0.542, *z* = 1.033, *P* = 0.302).

The GLMMs ranked with low AICs indicated that distance from the river mouth and ΣhTRS consistently negatively correlated to the density of eels, both of which were included in all candidate models with ∆AIC < 2, while velocity consistently positively correlated to the density of eels (Table 4). In addition to these three variables, the best model having lowest AIC value also selected depth and eel species as explanatory variables; however, the density of eels was significantly negatively correlated to only distance from the river mouth and ΣhTRS (Table 4; Fig. 2b,c).

**Table 4:** Akaike’s information criterion (AIC) ranking of the models that explain the density of anguillid eels (*A. japonica and A. marmorata*) in rivers of Japan (a), and coefficient values and associated probability of the best model (b).

(a)									
Rank	Explanatory variable								
	Depth	Distance from river mouth	Sediment	Eel species	Vegetation	Velocity	Trans-river structure	AIC	∆AIC
1	−0.013	−0.130		+		0.012	−0.046	313.9	0.000
2		−0.128				0.013	−0.047	314.4	0.428
3		−0.127		+		0.012	−0.048	314.4	0.484
4	−0.012	−0.136	+	+		0.009	−0.045	314.5	0.614
5		−0.134	+	+		0.010	−0.047	314.6	0.672
6	−0.011	−0.134				0.012	−0.044	314.6	0.679
7	−0.012	−0.118	+	+			−0.050	315.1	1.123
8		−0.136	+			0.011	−0.046	315.1	1.171
9		−0.115	+	+			−0.052	315.2	1.236
10	−0.013	−0.130		+	−0.182	0.011	−0.046	315.5	1.563
11	−0.010	−0.142	+			0.010	−0.043	315.7	1.728
12		−0.128			−0.228	0.012	−0.047	315.7	1.737
13		−0.127		+	−0.219	0.012	−0.048	315.8	1.840
**(b)**									
**Parameter**			**Estimate**		**Standard error**	***z*** **value**		***p*** **value**	
(Intercept)			−3.640		0.516	−7.049		0.000*	
Trans-river structure			−0.046		0.020	−2.254		0.024*	
Distance from river mouth			−0.128		0.057	−2.238		0.025*	
Depth			−0.013		0.009	−1.523		0.128	
Velocity			0.012		0.006	1.869		0.062	
Eel species_*A. marmorata*			0.930		0.554	1.679		0.093	

Plus and blank indicate significant or no significant effects of qualitative variable on the density of anguillid eels, respectively. ∆AIC indicates differences between AIC values of the best model (Rank 1) and selected model. The asterisks indicate statistical significant values.

Similarly, the optimal GLMMs showed that distance from the river mouth and ΣhTRS consistently negatively correlated to the number of other diadromous species, whilst velocity consistently positively correlated to the number of other diadromous species (Table 5). All these variables were included in all candidate models with ∆AIC < 2. The best model selected only these variables as explanatory variables, all of which were significantly correlated to the number of other diadromous species (Table 5; Fig. 2d,e,f).

**Table 5:** Akaike’s information criterion (AIC) ranking of the models that explain the number of non-anguillid eel diadromous species in rivers of Japan (a), and coefficient values and associated probability of the best model (b).

(a)								
Rank	Explanatory variable							
	Depth	Distance from river mouth	Sediment	Vegetation	Velocity	Trans-river structure	AIC	∆AIC
1		−0.048			0.008	−0.019	323.5	0
2		−0.044	+		0.009	−0.019	324.1	0.542
3		−0.048		0.058	0.009	−0.018	325.3	1.716
4	−0.002	−0.048			0.008	−0.019	325.3	1.729
**(b)**								
**Parameter**			**Estimate**	**Standard error**	***z*** **value**		***p*** **value**	
(Intercept)			1.767	0.105	16.811		0.0000*	
Trans-river structure			−0.019	0.005	−3.639		0.0003*	
Distance from river mouth			−0.048	0.014	−3.315		0.0009*	
Velocity			0.008	0.002	4.317		0.0000*	

Plus and blank indicate significant or no significant effects of qualitative variable on the density of anguillid eels, respectively. ∆AIC indicates differences between AIC values of the best model (Rank 1) and selected model. The asterisks indicate statistical significant values.

### Trophic level

**Figure 3:**
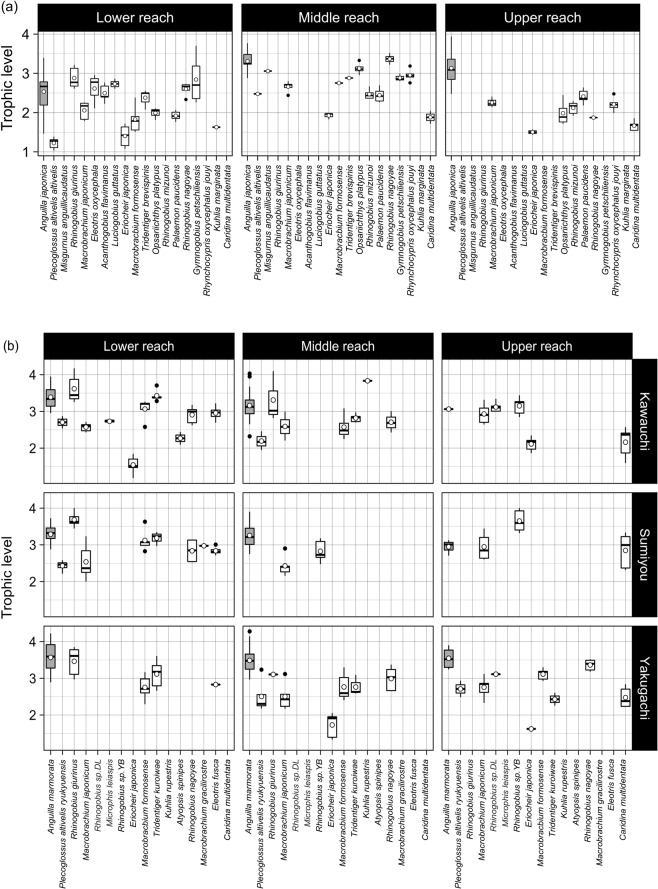
Trophic levels of all freshwater animals including *A. japonica* and *A. marmorata* collected in the Aono River watershed (**a**) and the three rivers of the Amami-Oshima Island (**b**), Japan, respectively. In the boxplots, the middle lines and open circles indicate the median and average, respectively, the boxes represents the 0.25 and 0.75 quartiles, the whiskers are the values that are within 1.5 of the interquartile range, and the dots show outliers. *A. japonica* and *A. marmorata* are shown in gray. The blanks indicate that the species were not collected.

The mean ± SD of TL of *A. japonica* was 2.9 ± 0.6 (2.5 ± 0.6, 3.3 ± 0.3 and 3.1 ± 0.5 at lower, middle and upper reaches in the Aono River, respectively; Fig. 3a). These values were significantly higher than those of other species in all reaches (Wilcoxon rank sum test, *p* < 0.05). Cliff’s delta statistic showed that the significant difference of TL between *A. japonica* and other species was found in the middle and upper reach of the river (Cliff’s delta statistic |d | > 0.33; Supplementary Table S3).

The mean TLs of *A. marmorata* collected in all reaches of each river in the Amami-Oshima Island were higher than 3, and the mean ± SD of all eels was 3.4 ± 0.4 (Fig. 3b). These values were significantly higher than those of other species in all reaches of each river, with exception of the upper reach of the Sumiyo River (Wilcoxon rank sum test, *p* < 0.05; Cliff’s delta statistic |d | > 0.33; Supplementary Table S3).

## Discussion

The present study tested the hypothesis of whether two eel species (*A. japonica and A. marmorata*) can act as surrogate species for conservation of freshwater biodiversity in Japanese rivers. The results characterized how eels represent freshwater biodiversity in our selected rivers. Our results showed that eels were the widest topographically-distributed species in the rivers, covering almost all of the sampling sites. This suggests that eels have the potential to be an indicator of river-ocean connectivity. This wide distribution of eels is consistent with the umbrella species concept that the presence of species with large area requirements will also encompass a whole suite of species with more limited spatial needs^13,18^. Additionally, the stable isotopic analyses supported their potential as an umbrella species; the TL of eels was greater than three – these values are normally associated with secondary consumers. These were higher than that of other species, indicating that they are likely one of the highest-order predators in freshwater ecosystems; their presence is indicative of healthy food web. It should be noted that the TLs of eels and their prey animals used in this study varied depending on the extent to which they relied on prey/food items other than those in the littoral food web. Moreover, the estimates of TL of animals depend strongly on the values of the TEF. In this study, TLs of eels were calculated by setting the TEF at 2.1‰ following Kaifu *et al*.^30^ that estimated the unique TEF value of reared yellow-phase Japanese eels. Kaifu *et al*.^30^ reported that TLs of wild Japanese eels estimated by stable isotope ratios using the unique TEF were consistent with results of their stomach contents^30^; thus using the unique TEF to estimate TLs of eels would be better than using the most general value from literatures (3.4‰)^50^. Eels are well known as a facultative polytrophic predator that feed on a range of species (*A. australis* and *A. dieffenbachii*^32^; *A. rostrata*^33^; *A. anguilla*^31^; *A. japonica*^29,30^), and their diets likely change in response to available prey^29,30^. Eels, therefore, need the presence of diverse lower TL animals for foods.

A significant positive relationship between the density of eels and the number of other diadromous species was found, indicating that density of eels may reflect the degree of a biodiversity in rivers. The optimal GLMMs, however, revealed that both the density of eels and the number of other diadromous species were significantly negatively related with distance from the river mouth and ΣhTRS, both of which are parameters relating to the river-ocean connectivity, although it should be noted that the effects of a high structure and low successive structures having same ΣhTRS values, on movement of eels were not discriminated by the models. These results imply that the observed positive relationship between eel density and the number of other diadromous species was likely an indirect relationship through the river-ocean connectivity, i.e. both eel density and number of other diadromous species were higher in sites where the river-ocean connectivity was better and vice versa. Diversity of diadromous species tends to be higher closer to the sea^58^. However, some diadromous species prefer upstream areas (e.g. *Sicyopterus japonicus*, Freshwater prawn *Macrobrachium japonicum*, Japanese marsh shrimp *Caridina multidentata*, and Japanese mitten crab *Eriocheir japonica*)^59–61^. Trans-river structures can prevent the movement of such species, leading to reduced diversity of diadromous species upstream. Accordingly, eel density is seemingly correlated with diversity of diadromous species affected by the river-ocean connectivity. Many studies have reported negative relationships between eel density and the distance from the river mouth^62–66^ and that trans-river structures limit distributions of diadromous species including eels by impeding their movements^37,67–72^, supporting our findings. Therefore, eels would be a suitable indicator of river-ocean connectivity, with functions as both a ‘health’ and ‘biodiversity’ indicator species^11^. Improving river-ocean connectivity for eels can also provide better connectivity for other freshwater animals.

Eels can also be an effective flagship species for aquatic conservation^24^. They are globally distributed, not only in entire river basins, as presented in this study, but also in the high seas, pelagic waters as deep as the bathypelagos, coastal marine waters, bays, lagoons, and brackish estuaries^28^. Moreover, eels have been a food resource for millennia and food cultures found across the world^73,74^. Today, they are commercially important fisheries species, and juvenile to large eels are harvested, farmed, traded and consumed on a global scale^75^. Eels are also culturally important and they appear in legends as objects of awe and respect^74^: for example, eels are a totem animal for some families and tribes in Micronesia; native Canadian, Māori and Aboriginal use eels ceremonially; eels can play an important role in myths in South East Asia; and in Japan, Buddhists in some villages never eat eels as they are messengers of a saint^73^. They have also entered our languages, prints, novels, tales and movies^73^: in Europe, eels appear in a children’s natural history book. All of these representations seem to be driven by their mysterious behavior. The distributional, commercial, and cultural importance of eels shows their value as a flagship species, with the ability to stimulate the public interest. As a taxa, eels can be indicator, umbrella and flagship species, and as such a comprehensive surrogate species for conservation of freshwater biodiversity.

This study was conducted in Japan, a part of islands formed by the accretionary prism, where diadromous species are dominant, however, there are 16 anguillid eel species and they distributed more broadly than this. Compared to the regions formed by the accretionary prism, rivers in the eroded regions and the craton have high diversity of the resident freshwater species^36^ and the effect of river-ocean connectivity on freshwater biodiversity of such areas is expected to be lower. Because trans-river structures would also prevent movement of resident freshwater species, eels might act as an indicator of riverine connectivity. Additionally, the topological distribution range, position in food web as a top-predator, commercial, and cultural importance of eels, are globally common. Therefore, eels could act as a surrogate species for conservation of freshwater biodiversity irrespective of geological terrain. Future studies conducting surveys throughout the distributional range of anguillid eel species are required to test this hypothesis.

Conserving eels should relate strongly to the conservation of freshwater biodiversity if the umbrella species concept is applicable. Given the wide distribution of eels in rivers, conserving even a proportion of these the large areas would protect other sympatric species. As eels are one of the highest-order predators in freshwater ecosystems, suitable habitats require the presence of diverse lower TL animals for foods. Indeed, a recent study reported that the collapse of prey species was correlated with the decline of *A. japonica* abundance in a Japanese lake^76^, suggesting the importance of the presence of diverse lower TL animals.

Restoring and maintaining river-ocean connectivity would conserve eels as well as biodiversity in freshwater ecosystems more broadly. In this study, the density of eels was negatively related with both distance from the river mouth and ΣhTRS, which was consistent with results of previous studies for *A. japonica*^77^. Although some eels could likely pass trans-river structures by climbing them vertically^78^, it has been reported that the structures decrease eel density by inhibiting their upstream migration^67,68,70^. According to the AIC values of models in this study, the relationship between the density of eels and ΣhTRS increased in strength when ΣhTRS was calculated by considering all heights of the structure. As many low-height trans-river structures were found in the study rivers (median = 1.0 m), our findings suggest that trans-river structures can curtail eel movements regardless of its height. In addition to the height of trans-river structure, their design e.g., slope, material, the presence/absence of fish passage, location in the watershed etc. may also influence whether eels can pass the structure. Despite these other factors being absent from the analysis, our models showed that eel density was negatively correlated with ΣhTRS, indicating that structures found in this study can impede the passing of eels. By incorporating additional factors to future models, it is expected that a more robust understanding of the correlation between eel density and ΣhTRS can be identified. The habitat loss that was resulted from the barriers has been identified as a major impact on eels species^71,72,79,80^. The construction of hydropower dams during the twentieth century in the St. Lawrence catchment in Canada caused a 40% habitat loss for *A. rostrata* in this basin^81^. This situation is similar or worse in the United States^82^. In Europe, 50–90% of available freshwater habitat was lost by the end of the twentieth century^72,79^. For *A. japonica*, approximately 75% of effective habitat was lost between the 1970s and 2010 in East Asia^71^. Therefore, reducing the impacts of barriers and increasing access to suitable habitats could lead to increased local abundance of eels, and potentially biodiversity in freshwater ecosystem.

In this study, we demonstrated that eels have the potential to be a surrogate species for conservation of freshwater biodiversity in Japan, a part islands formed by the accretionary prism. Surrogate species may present useful strategies for conservation planning but must be carefully evaluated to ensure their proper use.

## Supplementary information

Supplementary Information.

## Data Availability

The data that support the findings of this study are available in the Supporting Information File.
