# Supplementary material for: Anguillid eels as a surrogate species for conservation of freshwater biodiversity in Japan
Source: Sci Rep. 2020 May 29;10:8790. doi: 10.1038/s41598-020-65883-4 (PMC7260186; doi:10.1038/s41598-020-65883-4)
Supplement: Supplementary file 1 — Supplementary Information. [file 41598_2020_65883_MOESM1_ESM.pdf]

Supplementary Information

Anguillid eels as a surrogate species for conservation of freshwater biodiversity in Japan

Hikaru Itakura, Ryoshiro Wakiya, Matthew Gollock, Kenzo Kaifu

Corresponding Author
Hikaru Itakura
University of Maryland

Table S1 Environmental conditions of sampling sites of freshwater species.

River	Width (m)	Area (m^2)	Depth (cm)	Velocity (cm s^{-1})
Hatauchi	4.9 ± 1.5 (3.1–8.6)	200 ± 43 (163–286)	29 ± 8 (21–44)	51 ± 14 (32–69)
Aono	8.4 ± 9.8 (1.4–56)	99 ± 34 (30–222)	36 ± 15 (8–71)	32 ± 26 (6–119)
Kaizoko	5.1 ± 1.7 (3–8.1)	203 ± 61 (137–330)	31 ± 12 (13–45)	22 ± 11 (5–43)
Yakugachi	14.4 ± 10 (5.4–36)	155 ± 70 (83–291)	42 ± 15 (21–69)	45 ± 24 (7–79)
Sumiyo	16.6 ± 10.2 (3.7–37.7)	129 ± 52 (87–245)	38 ± 11 (21–55)	17 ± 8 (6–34)
Kawauchi	8.7 ± 5 (2.7–18)	128 ± 46 (67–217)	46 ± 18 (26–73)	30 ± 16 (5–54)

Each value are shown by mean ± SD and range (in parentheses).

Table S2 Freshwater species collected in the Mainland Japan and the Amami-Oshima Island, Japan.

Region	Species	Category	Migratory type
Mainland Japan	*Anguilla japonica*	Fish	Diadromous
Mainland Japan	*Plecoglossus altivelis altivelis*	Fish	Diadromous
Mainland Japan	*Carassius* sp.	Fish	Non-diadromous
Mainland Japan	*Candidia temminckii*	Fish	Non-diadromous
Mainland Japan	*Opsariichthys platypus*	Fish	Non-diadromous
Mainland Japan	*Rhynchocypris oxycephalus jouyi*	Fish	Non-diadromous
Mainland Japan	*Rhychocyris Iagowskii steindachneri*	Fish	Non-diadromous
Mainland Japan	*Misgurnus anguillicaudatus*	Fish	Non-diadromous
Mainland Japan	*Cobitis* sp. BIWAE type B	Fish	Non-diadromous
Mainland Japan	*Mugil cephalus*	Fish	Diadromous
Mainland Japan	*Kuhlia marginata*	Fish	Diadromous
Mainland Japan	*Rhyncopelate oxyhynchus*	Fish	Diadromous
Mainland Japan	*Acanthopagrus schlegelii*	Fish	Diadromous
Mainland Japan	*Eleotris oxycephala*	Fish	Diadromous
Mainland Japan	*Luciogobius guttatus*	Fish	Diadromous
Mainland Japan	*Sicyopterus japonicus*	Fish	Diadromous
Mainland Japan	*Tridentiger obscurus*	Fish	Diadromous
Mainland Japan	*Tridentiger brevispinis*	Fish	Diadromous
Mainland Japan	*Rhinogobius nagoyae*	Fish	Diadromous
Mainland Japan	*Rhinogobius giurinus*	Fish	Diadromous
Mainland Japan	*Rhinogobius mizunoi*	Fish	Diadromous
Mainland Japan	*Rhinogobius fluviatilis*	Fish	Diadromous
Mainland Japan	*Rhinogobius brunneus*	Fish	Diadromous
Mainland Japan	*Gymnogobius petschiliensis*	Fish	Diadromous
Mainland Japan	*Acanthogobius flavimanus*	Fish	Diadromous
Mainland Japan	*Redigobius bikolanus*	Fish	Diadromous
Mainland Japan	*Cottus kazika*	Fish	Diadromous

Mainland Japan	*Takifugu niphobles*	Fish	Diadromous
Mainland Japan	*Caridina multidentata*	Crustacean	Diadromous
Mainland Japan	*Caridina typus*	Crustacean	Diadromous
Mainland Japan	*Caridina leucosticta*	Crustacean	Diadromous
Mainland Japan	*Palaemon paucidens*	Crustacean	Diadromous
Mainland Japan	*Macrobracbium formosense*	Crustacean	Diadromous
Mainland Japan	*Macrobrachium japonicum*	Crustacean	Diadromous
Mainland Japan	*Metapenaeus ensis*	Crustacean	Diadromous
Mainland Japan	*Geothelphusa dehaani*	Crustacean	Non-diadromous
Mainland Japan	*Eriocheir japonica*	Crustacean	Diadromous

Table S2 continued

Region	Species	Category	Migratory type
Amami-Oshima Island	*Anguilla marmorata*	Fish	Diadromous
Amami-Oshima Island	*Plecoglossus altivelis ryukyuensis*	Fish	Diadromous
Amami-Oshima Island	*Microphis leiaspis*	Fish	Diadromous
Amami-Oshima Island	*Kuhlia rupestris*	Fish	Diadromous
Amami-Oshima Island	*Kuhlia marginata*	Fish	Diadromous
Amami-Oshima Island	*Eleotris fusca*	Fish	Diadromous
Amami-Oshima Island	*Sicyopterus japonicus*	Fish	Diadromous
Amami-Oshima Island	*Tridentiger kuroiwae*	Fish	Diadromous
Amami-Oshima Island	*Rhinogobius nagoyae*	Fish	Diadromous
Amami-Oshima Island	*Rhinogobius giurinus*	Fish	Diadromous
Amami-Oshima Island	*Rhinogobius brunneus*	Fish	Diadromous
Amami-Oshima Island	*Rhinogobius* sp. YB	Fish	Non-diadromous
Amami-Oshima Island	*Rhinogobius* sp. DL	Fish	Diadromous
Amami-Oshima Island	*Caridina multidentata*	Crustacean	Diadromous
Amami-Oshima Island	*Atyopsis spinipes*	Crustacean	Diadromous
Amami-Oshima Island	*Macrobrachium japonicum*	Crustacean	Diadromous
Amami-Oshima Island	*Macrobracbium formosense*	Crustacean	Diadromous

Amami-Oshima Island	*Macrobrachium lar*	Crustacean	Diadromous
Amami-Oshima Island	*Macrobrachium gracilirostre*	Crustacean	Diadromous
Amami-Oshima Island	*Geothelphusa obtusipes*	Crustacean	Non-diadromous
Amami-Oshima Island	*Eriocheir japonica*	Crustacean	Diadromous
Amami-Oshima Island	*Hemigrapsus penicillatus*	Crustacean	Diadromous

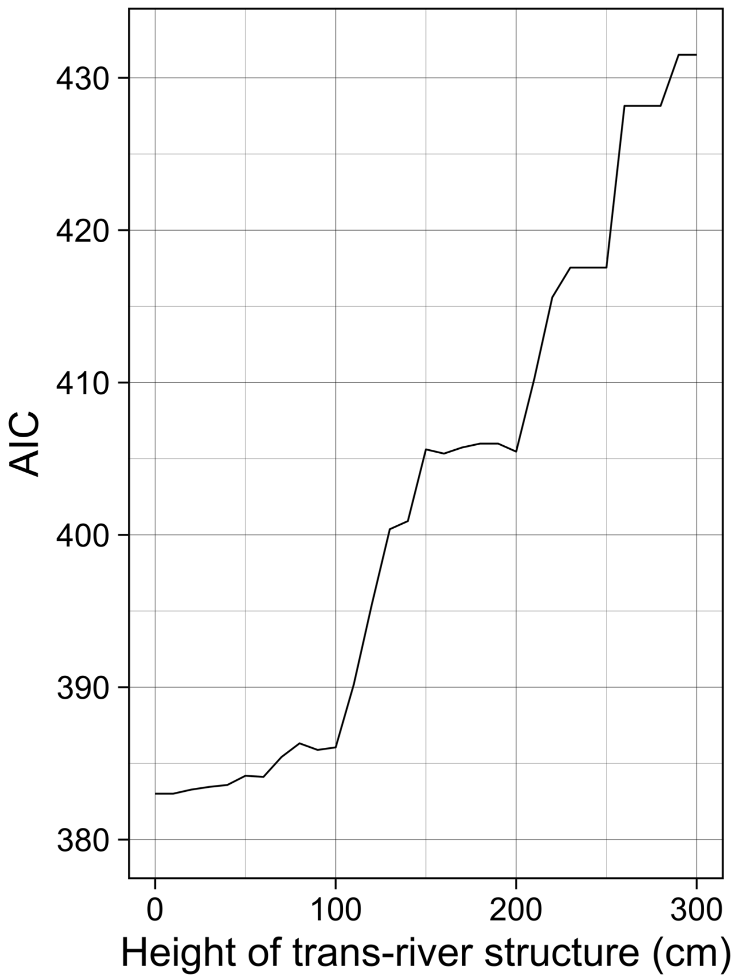

Figure S1 Comparison of Akaike's information criterion (AIC) for candidate models of density of anguillid eels using the different lower limit value of the height of trans-river structure that should be included in the calculation of cumulated height of the structure.

Table S3 Statistical comparisons of trophic level (TL) between anguillid eels and other freshwater species along the reaches of each river, based on Wilcoxon rank sum test and Cliff's delta statistic (|d|).

River	Reach	No. of speices	n		Wilcoxon rank sum test		Cliff's delta statistic (|d|)	95% CI
			eels	other species	*w*	*p*		
Aono	Lower	15	16	62	659	0.044	0.328 (Small)	0.000 to 0.592
	Middle	14	9	41	321	0.000	0.737 (Large)	0.444 to 0.888
	Upper	9	6	31	183	0.000	0.968 (Large)	0.818 to 0.995
Kawauchi	Lower	11	30	42	1014	0.000	0.610 (Large)	0.389 to 0.764
	Middle	8	18	23	317	0.003	0.531 (Large)	0.206 to 0.751
	Upper	6	1	25	-	-	0.360 (Medium)	-
Sumiyo	Lower	9	14	32	330	0.011	0.471 (Medium)	0.174 to 0.689
	Middle	3	7	10	63	0.005	0.800 (Large)	0.342 to 0.951
	Upper	4	3	15	16	0.498	-0.289 (Small)	-0.592 to 0.086
Yakugachi	Lower	5	10	16	126	0.014	0.575 (Large)	0.124 to 0.829
	Middle	8	27	30	765	0.000	0.889 (Large)	0.736 to 0.956
	Upper	9	4	26	97	0.003	0.865 (Large)	0.541 to0.966

Effects values are classified into “negligible” ($|d| < 0.147$), “small” ($|d| < 0.330$), “medium” ($|d| < 0.474$) and “large” ($|d| > 0.474$) (Romano et al. 2006).

Reference

Romano J, Kromrey JD, Coraggio J, Skowronek J. 2006. Appropriate statistics for ordinal level data: Should we really be using t-test and Cohen’s d for evaluating group differences on the NSSE and other surveys. Annual Meeting of the Florida Association of Institutional Research.
